# SARS-CoV-2 infection as possible downstream disease precipitator in autoantibody-positive insulin-dependent diabetes mellitus: a case report

**DOI:** 10.1186/s13052-022-01226-5

**Published:** 2022-02-23

**Authors:** Riccardo Schiaffini, Andrea Campana, Annalisa Deodati, Emanuela Peschiaroli, Maria Francesca Lanzillotta, Alessandra Fierabracci

**Affiliations:** 1 Diabetes and Growth Disorders Unit, Academic Department of Pediatrics, Bambino Gesù Children’s Hospital IRCCS, Rome, Italy; 2 Multispecialistic Pediatric Unit, Emergency Acceptance and General Pediatrics Department, Bambino Gesù Children’s Hospital, Rome, Italy; 3 Infectivology and Clinical Trials Research Department, Bambino Gesù Children’s Hospital IRCCS, Rome, Italy

**Keywords:** Type 1 diabetes, COVID-19, Disease etiopathogenesis, Management

## Abstract

**Background:**

SARS-CoV-2 causes lesions, in addition to lung, in endocrine organs such as the pancreas through ACE2 receptor. Recently the relationship between SARS-CoV-2 exposition and the incidence or evolution of clinical autoimmune diabetes has attracted the attention of diabetologists.

**Case presentation:**

We report the analysis of the clinical history of a child diagnosed for insulin-dependent diabetes mellitus (Type 1 diabetes) at the time a paucisymptomatic COVID-19 infection occurred, followed by well-controlled metabolic status. As opposite to previous findings SARS-CoV2 did not cause ketosis and ketoacidosis. Polydipsia was reported a few months and weight loss 4 weeks before SARS- CoV-2 infection suggesting that SARS-CoV-2 could not be the trigger of Type 1 diabetes in this patient.

**Conclusions:**

SARS-CoV-2 in this patient was an unexpected event in the course of disease. We advance the hypothesis that the SARS-CoV-2 infection, even if paucisymptomatic could have acted in the present case report as a hypothetical downstream precipitating factor; whilst the inciting triggering event of the autoimmune disease, as confirmed by the presence of circulating autoantibodies, could have occurred even before, as generally assumed for this category of disorders. The precipitating mechanism could have been the acute interaction between virus and the ACE receptor on the beta cells, at the time that hyperglycemia and glycosuria were ascertained, and HbA1c levels confirmed a metabolic dysregulation over the previous 3 months in absence of ketoacidosis.

## Background

The ongoing COVID-19 pandemic is caused by the severe acute respiratory syndrome- coronavirus-2 (SARS-CoV-2) which can lead to severe illness and death [1]. SARS-CoV-2 causes lesions not only in the lungs but also in other organs, including endocrine ones [1, 2]. Viral tropism in these tissues relies on receptors for the coronavirus spike protein, such as the angiotensin-converting enzyme 2 (ACE2) and the transmembrane serine protease 2 (TMPRSS2) [1]. Indeed SARS-CoV-2 infects and replicates within the cells of exocrine pancreas and pancreatic islets [2]. SARS-CoV-2 might therefore affect the function of beta cells and their insulin secretion thus contributing to metabolic dysregulation in COVID-19 patients demonstrated by increased hyperglycemia in Type 2 diabetes patients [2] and ketoacidosis occurring in diabetic and non-diabetic individuals [3]. Furthermore, the relationship between ketoacidosis and the onset Type 1 diabetes (T1D), in absence of islet- related autoantibodies (Abs), has been reported [4].

It is hypothesized that SARS-CoV-2, like other viruses or unknown environmental factors, could indeed act as a trigger for the onset of autoimmunity [5]. Some patients have developed Guillain–Barré syndrome or systemic lupus erythematosus, autoimmune haemolytic anaemia, thrombotic thrombocytopenic purpura or thrombocytopenic purpura and Graves’ disease after COVID-19 infection [6, 7]. Nevertheless the role of SARS-CoV-2 as trigger factor for autoimmunity needs still to be fully unraveled. We present a case report of a child affected by T1D with a paucisymptomatic COVID-19 infection that offers the opportunity to speculate on the possible mechanism of the viral influence on the disease.

## Case presentation

We present the case of a 12 year old white male patient with height 156 cm and weight 53.5 kg. He was admitted to our emergency Department with a history of headaches and fever over the past 3 days, responsive to paracetamol and corticosteroid treatment, without the typical chest pain. In the previous 3 months the patient presented polydipsia and experienced a weight loss of 5 kg in the past 4 weeks. The family history was negative for autoimmune diseases but revealed an aunt on the mother’s side with Friedreich ataxia and hypertension. Laboratory testing demonstrated hyperglycemia (> 400 mg/dl, normal range 60–100) with c-peptide level 2.47 ng/mL (normal range 1.10–4.40) and haemoglobin A1c (HbA1c) of 122 mmol/mol (> 48 decisional level for diabetes diagnosis), without ketoacidosis (ph 7.34, BE -5.8 HCO3 std 19.5). In consideration of the acute onset, the clinical phenotype and the age of the child, T1D was assumed. Human leukocyte antigen (HLA) genotyping revealed that the patient had the predisposing haplotypes DRB1*03*, DQB1*02* and DQA1*05*. Celiac disease haplotype 1 DRB1*03-DQA1*05-DQB1*02 and haplotype 2 DRB1*07-DQA1*0201-DQB1*02 were also detected. Serum diabetes-related glutamic acid decarboxylase isoform 65 (GAD65) Abs and anti-zinc transporter 8 (ZnT8) Abs tested positive, while tyrosine phosphatase-insulinoma-associated antigen (IA2), insulin (AIA), thyroperoxidase (TPO), thyroglobulin (Tg) and transglutaminase (ATG) Abs were negative (Table 1). At referral, COVID-19 infection was confirmed by molecular analysis of naso-pharyngeal and ocular swabs. Infection by other respiratory viruses, including Adenovirus, influenza A/B, parainfluenza 1–4, syncytial A/B, Metapneumovirus, Coronaviruses OC43, 229E, NL63, Rhinovirus A/B/C, Bocavirus 1/2/3/4 and Enterovirus, was excluded. He did not present metabolic comorbidities and markers (no hypertension, negative CRP (0.5 mg/dl), HdlC (25 mg/dl), LdlC (52 mg/dl), triglycerides (131 mg/dl), normal ALT (14 U/L), AST (18U/L), gGT (7 u/L) and ferritin (119 ng/ml) levels, no liver steatosis).

**Table 1:** Autoantibodiesscreening in the serum of case reports. The Table refers to levels of islet-related, thyroid-related, ATG serum autoantibodies and EMA for Patient 1 and 2

Reference range		Patient 1	Patient 2
*GAD65-Ab*	Negative < 5 Positive > 5	34 (U ml^−1^)	91 (U ml^−1^)
**IA-2-Ab**	Negative < 7.5 Positive > 7.5	< 7.5 (U ml^−1^)	134 (U ml^−1^)
**ZnT8-Ab**	Negative < 15 Positive > 15	642 (U ml^−1^)	< 15 (U ml^−1^)
**AIA-Ab**	Negative < 10 Borderline > 7–10 Positive > = 10	< 10 (U ml^−1^)	NT
*TPO*	< 34	< 9.0 (IU/mL)	10.9 (U ml^−1^)
*Tg*	< 115 IU/mL	Negative	Negative
*EMA IgA*	Negative	NT	weakly positive
*EMA IgG*	Negative	NT	NT
**ATG IgA**	Negative = < 20 weakly positive = 20–30 Positive = > 30	8.3 (CU)	530.9 (CU)

*NT *not tested

**Fig. 1:**
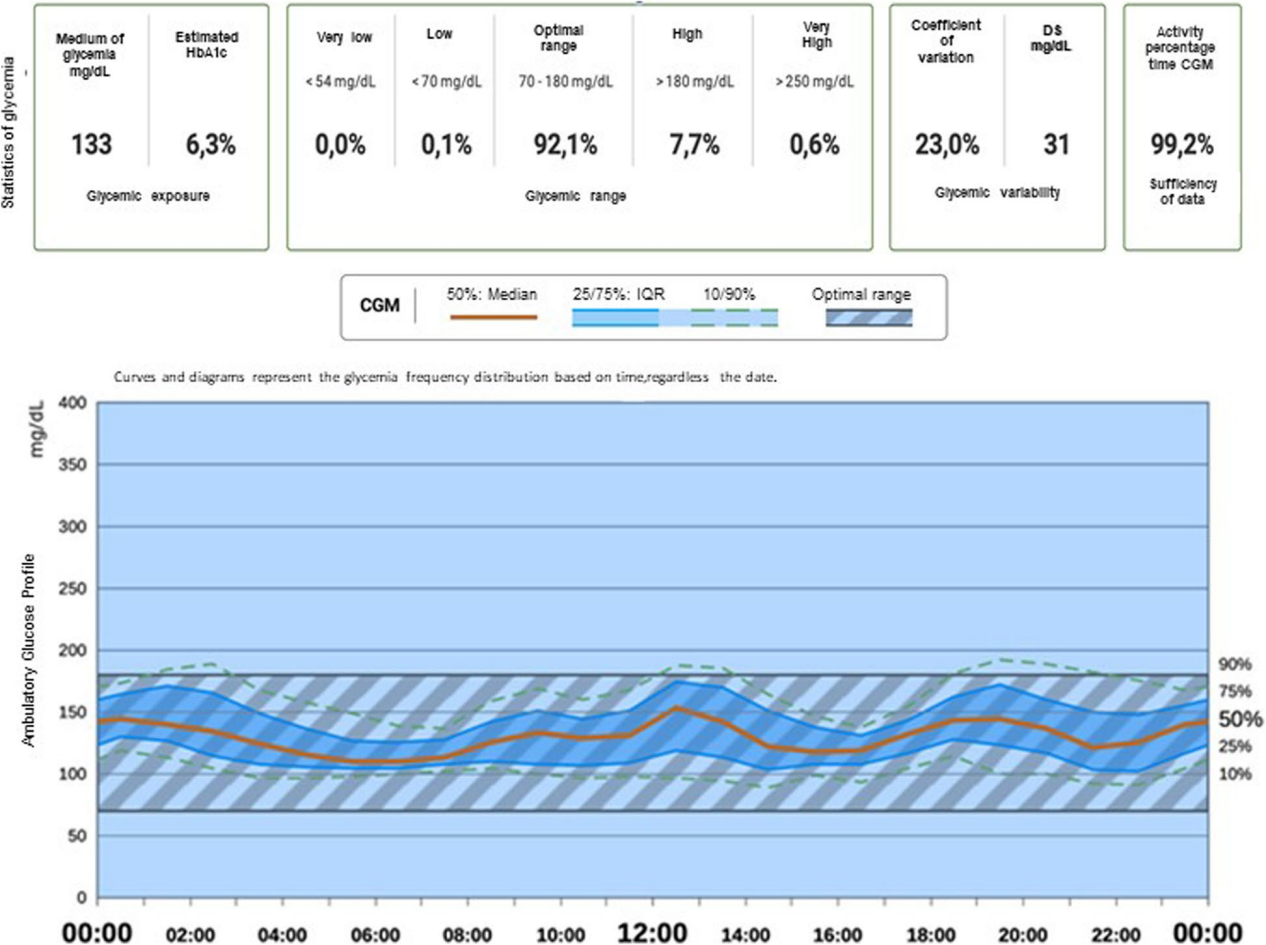
Average glucose trend and metrics as shown by the CGM for patient 1

Lipase, pancreatic amylase and TSH were normal. There was no evidence of retinopathy, nephropathy or of exocrine pancreas involvement since the patient did not complain of diarrhea, increased stool volume and/or fatty stools. The patient was promptly administered subcutaneous insulin therapy. Over the course of the following few days, his blood glucose levels stabilized. The patient received an educational programme with guidelines for Type 1 diabetes management and was discharged from the hospital in good conditions after 10 days. Four months from the onset, the patient has a well-controlled metabolic status, with HbA1c value of 46 mmol/mol, following a classical basal-bolus insulin scheme with Multi—Daily Injections, with a total insulin requirement of 0.5 UI/kg/die. The patient also began to use a Continuous Glucose Monitoring (CGM) system in order to improve blood glucose monitoring and consequently minimize hypo and hyperglycemic episodes, promoting good glucose control with a Time in the ideal (70–180 mg/dl) glucose Range (TIR) of 92% (Fig. 1).

## Discussion

Recently the relationship between SARS-CoV-2 exposition and the incidence or evolution of clinical autoimmune diabetes has attracted the attention of diabetologists based on the hypothesis that diabetes may occur as an acute complication following SARS-CoV-2 infection at least in predisposed subjects. Recent investigations suggest that the occurrence of infection in pediatric patients affected by autoimmune diseases does not seem to affect the severity and evolution of the clinical manifestations or the management of the autoimmune disease [8].

The medical history of the patient presented here confirms the recent clinical experiences of pediatric diabetologists, in which SARS-CoV-2 infection does not appear to cause persistent glycemic decompensation in children and adolescents with T1D [8]. Moreover, young patients with disease onset most often have asymptomatic or paucisymptomatic SARS- CoV-2 infection. On a general ground, people with diabetes do not exhibit increased susceptibility to SARS-CoV-2 infection [8].

It is, however, of interest to remark that in the clinical history of the patient reported here the autoimmune process preceded the clinical manifestations of T1D with the symptom of polydipsia, occurring a few months before COVID-19 infection, which was paucisymptomatic. This was also observed in another 8 year old male referred to our Hospital with T1D onset manifested with polyuria and polydipsia in the absence of ketoacidosis associated to celiac disease (Table 1). We confirm that the management of the disease in the patient so far was not significantly affected, being that presently his glycemic status is controlled by the established insulin administration regiment.

Based on previous epidemiological observations, a few months generally lapse between a triggering unknown event, including stress, infectious agents or hormonal perturbations, and the onset of an autoimmune clinical disease [5]. T1D is consequently caused by primary mediators, i.e. activated autoreactive T cells, that invade the pancreatic islets leading to insulitis. During the long-preclinical period that precedes the disease onset and often lasts for years [9], the insulitis would remain innocuous until incited to destruction by any possible secondary event, i.e. the systemic or local reactivity to a viral superantigen [10]. Activated T cells at this stage expand and destroy insulin-producing beta cells as they react to pancreatic antigens. As a general effect, infectious agents may enhance self-antigen presentation, lead to the involvement of different autoantigens or of different epitopes of the same protein [10].

In light of the foregoing, we advance the hypothesis that the SARS-CoV-2 infection, even if paucisymptomatic could have acted in the present case report as a downstream precipitating factor; whilst the inciting autoimmune triggering event leading to insulitis, as confirmed by the presence of circulating autoantibodies, could have occurred even before, as generally assumed for this category of disorders [5]. SARS-CoV-2 infection could have precipitated the clinical disease onset through the acute interaction between the virus and the ACE receptor on the beta cells leading to massive destruction of their reservoir already compromised by the insulitis previously evoked by the triggering agent. Whilst the putative contribution of the virus on the activation of infiltrating T cells remains to be demonstrated.

Indeed SARS-CoV-2 infection occurred in our patient at the time that hyperglycemia and glycosuria were ascertained, and HbA1c levels confirmed a metabolic dysregulation over the previous 3 months in absence of ketoacidosis.

## Conclusions

Firstly SARS-CoV-2 infection has effect on T1D clinical onset. Overt disease manifested in this patient at the time that paucisymptomatic viral infection occurred. Secondly, SARS-CoV-2 could not be the trigger of autoimmunity in T1D. Polydipsia was reported a few months and weight loss 4 weeks before SARS-CoV-2 infection. Thirdly, SARS-CoV-2 would have acted as downstream disease precipitator. The direct destruction of beta cells following ACE2 receptor binding is putatively involved. Fourthly, as opposite to previous findings SARS-CoV-2 did not cause ketosis and ketoacidosis.

## Data Availability

Data sharing is not applicable to this article as no datasets were generated or analysed during the current study.

## Abbreviations

Abs: Autoantibodies
ACE2: Angiotensin-converting enzyme 2
AIA: Anti-insulin antibodies
ALT: Alanine aminotransferase
AST: Aspartate aminotransferase
ATG: Transglutaminase
CGM: Continuous Glucose Monitoring
COVID-19: Coronavirus disease 19
CRP: C-reactive protein
SARS-CoV-2: Severe acute respiratory syndrome-coronavirus 2
T1D: Type 1 diabetes
GAD65: Glutamic acid decarboxylase isoform 65
Ggt: Gamma-glutamyl transferase
HbA1c: Haemoglobin A1c
HdlC: High-density lipoprotein cholesterol
HLA: Human leukocyte antigen
IA2: Tyrosine phosphatase insulinoma-associated antigen
LdlC: Low-density lipoprotein cholesterol
TMPRSS2: Transmembrane serine protease 2
Tg: Thyroglobulin
TIR: Time in the ideal glucose Range
TPO: Thyroperoxidase
TSH: Thyroid- stimulating hormone
ZnT8: Zinc transporter 8
